# Significance of Zn Complex Concentration on Microstructure Evolution and Corrosion Behavior of Al/WS_2_

**DOI:** 10.3390/molecules28217290

**Published:** 2023-10-27

**Authors:** Pratiksha P. Gawas, Praveenkumar Pandurangan, Marzieh Rabiei, Arvydas Palevicius, Andrius Vilkauskas, Giedrius Janusas, Mozhgan Hosseinnezhad, Reza Ebrahimi-Kahrizsangi, Sohrab Nasiri, Jean Michel Nunzi, Venkatramaiah Nutalapati

**Affiliations:** 1 Functional Materials Laboratory, Department of Chemistry, Faculty of Engineering and Technology, SRM Institute of Science and Technology, SRM Nagar, Kattankulathur 603203, India; pratiksha.gws@rediffmail.com (P.P.G.); pp9547@srmist.edu.in (P.P.); 2 Faculty of Mechanical Engineering and Design, Kaunas University of Technology, Studentu Street 56, LT 51373 Kaunas, Lithuania; rabieimarzieh7@gmail.com (M.R.); arvydas.palevicius@ktu.lt (A.P.); andrius.vilkauskas@ktu.lt (A.V.); giedrius.janusas@ktu.lt (G.J.); 3 Department of Organic Colorants, Institute for Color Science and Technology, Tehran 1668836471, Iran; hosseinnezhad-mo@icrc.ac.ir; 4 Advanced Materials Research Center, Department of Materials Engineering, Najafabad Branch, Islamic Azad University, Najafabad Isfahan 85141-43131, Iran; rezaebrahimi@iaun.ac.ir; 5 Faculty of Mechanical Engineering, Optical Measurement Laboratory, Kaunas University of Technology, Studentu Street 56, L-116, LT 51373 Kaunas, Lithuania; 6 Department of Physics, Engineering Physics & Astronomy, Queens University, Kingston, ON K7L-3N6, Canada; nunzijm@queensu.ca

**Keywords:** composite, Al/WS_2_, ZnTerp-2TH, hydrothermal method, corrosion

## Abstract

Corrosion is a harmful processes which by definition is a chemical or electrochemical reaction between a substance (usually a metal) and the environment which leads to a change in the properties of the substance and has destructive effects. In this study, new composites consisting of Al/WS_2_/ZnTerp-2TH with 5 and 10 wt.% ZnTerp-2TH were prepared and the results were fully compared. Al/WS_2_ played the role of matrix and ZnTerp-2TH played the role of reinforcement. In other words, as a novelty to prevent the corrosion of Al/WS_2_, ZnTerp-2TH is designed and synthesized and showed good results when the corrosion ratio was reduced by the existence of ZnTerp-2TH. Furthermore, the NMR and mass analysis of ZnTerp-2TH were carried out, and the thermal properties, X-ray diffraction, Fourier-transform infrared (FTIR) spectroscopy, morphology, energy-dispersive X-ray spectroscopy (EDX) analysis and corrosion behavior of the composites were also discussed in detail. The crystal size values of composites were calculated by the modified Scherrer method 34, 26 and 27 nm for Al/WS_2_, Al/WS_2_/5 wt.% ZnTerp-2TH and Al/WS_2_/10 wt.% ZnTerp-2TH, respectively. The microstructural examination of the specimens showed that the reinforcing phase (ZnTerp-2TH) has a favorable distribution on the surface of Al/WS_2_ when it covers the cracks and holes. In addition, the corrosion investigation results showed that the addition of ZnTerp-2TH to Al/WS_2_ can improve the corrosion resistance when the E_corr_ and I_corr_ values of Al/WS_2_/10 wt.% ZnTerp-2TH were recorded in tandem −724 mV/decade and 5 uA cm^−2^.

## 1. Introduction

Recently, due to its high stability, more attention has been paid to composites based on metal sulfide materials to prevent contaminated environments. Among metal oxide materials, alumina is a solid base component, especially as a cathode layer in semiconductor photocatalysts, because it is considered readily available, cheap, with a high band gap around 5.67 eV and high reactivity [1]. However, the biggest problem is the corrosion of aluminum composites, which are very sensitive to oxygen [2]. Various mechanisms for the strength of a portion of the particles in the metal field are presented, including charge transfer from the matrix to the particles, the effect of grain size reduction (Hall–Petch equation) and the incompatibility of the coefficient of thermal expansion [3]. To overcome this limitation, numerous studies have been carried out, including doping complexes or dyes [4,5]. One of the parameters to decorate the structure and extend the absorption band edge of aluminum into the visible light region is the use of certain metal sulfides with ideal optical properties such as tungsten sulfide (WS_2_), which has the potential to transfer electrons to aluminum [6]. In each part of the WS_2_ structure, the atoms are covalent and each side is connected by van der Waals bonds [7]. The S-W-S plane can be connected via a weak Van der Waals force, and due to the indirect band gap of 1.4 eV, it can provide a small visible absorption region when connected to Al as a composite when the photoinduced electrons cannot effectively migrate to aluminum dioxide because the conduction band edge of WS_2_ is lower than that of aluminum dioxide [8]. Al-WS_2_ composites can be used in optical and aerospace industries if the corrosion and tribology issues are considered. Therefore, attention to corrosion protection of this kind of composite is very important. Ning Li et al. investigated the hall mobility of Al-doped WS_2_ films and the optical and electrical properties of the films prepared by atomic layer deposition and vulcanization [6]. Monica Ratoi et al. investigated the tribological properties of self-lubricating Al-WS_2_ composites [9]. In another study on Al-WS_2_ composites, Alla Zak et al. demonstrated that WS_2_ can prevent dislocation formation and improve physical properties [10]. Dapeng Li et al. improved the optical and electrical properties of Al-WS_2_ films by H_2_S sulfurization [11]. It is worth noting that one of the best derivatives to simultaneously improve the corrosion composition of Al-WS_2_ is the use of Zn complexes. Zinc complexes have lower costs as well as better performance compared to titanium dioxide for destruction due to the bond gap energy of 3.3 eV; these complexes, stimulated by radiation and by transferring electrons, can remove several organic pollutants in both basic and acidic environments [12,13,14,15]. Taking into account these advantages, Zn complexes are an ideal choice for preventing the degradation of organic dyes. Moreover, tuning the morphology and manipulating the surface can be an attractive and effective strategy to optimize the corrosion performances.

Heterocyclic organic compounds are considered the most efficient inhibitors for metals in acidic mediums because of their molecular structure, which includes nitrogen, phosphorus, oxygen, sulfur, electronegative functional groups and p-electrons in triple or conjugated double bonds, as well as aromatic rings [16,17,18,19]. The effectiveness of organic compounds in preventing corrosion is determined by their physicochemical properties, such as their electronic structure, functional groups, steric effects, the ability to donate electrons through p-orbitals and the electronic density of donor atoms. When these inhibitors come into contact with metal surfaces, their lone pairs and/or p-orbitals interact with the d-orbitals of the metal atoms, resulting in the formation of a protective film that acts as a barrier [20]. Thiohydantoins are the sulfur analogs of hydantoins which are formed by substituting one or both of the carbonyl groups with thiocarbonyl groups [21]. Notably, 2-thiohydantoins (2TH), among the known thiohydantoins, exhibit corrosion inhibitory properties [16]. Additionally, the terpyridine derivatives are widely recognized and acknowledged for their exceptional chelating properties. Their versatility as ligands enables precise control over metal coordination, facilitating advances in catalysis, sensing, materials science and supramolecular chemistry [22].

**Figure 1:**
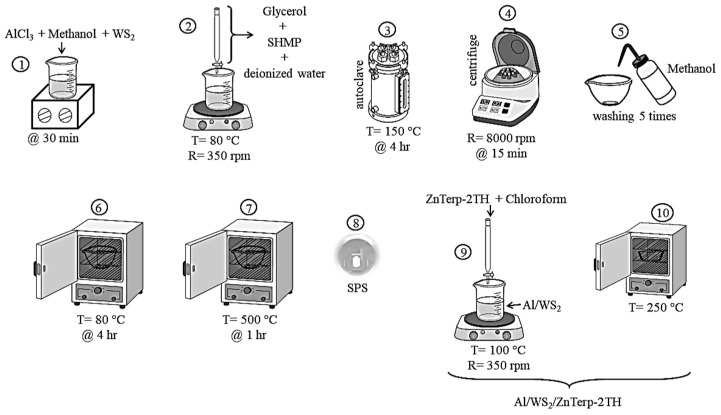
The synthesis route of Al/WS_2_ and Al/WS_2_/ZnTerp-2TH composites.

Therefore, we report here the preparation of Al/WS_2_/ZnTerp-2TH, where ZnTerp-2TH serves as a reinforcement because it reduces the corrosion ratio. In this study, the characterization of these composites composed of 5 and 10 wt.% of ZnTerp-2TH is discussed in detail because the high stability and recyclable use of the composite enable the use of industrial applications to improve the corrosion parameters. The chemical and synthesis pathway of ZnTerp-2TH is presented in Figure 1.

## 2. Results and Discussion

### 2.1. NMR and Mass Analysis of ZnTerp-2TH

**Scheme 1:**
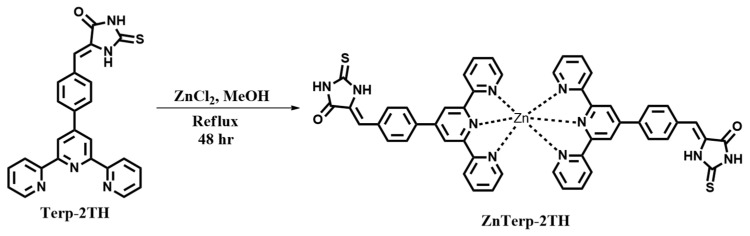
Synthetic route for ZnTerp-2TH.

Terp-2TH was synthesized according to the literature-reported procedure [23,24]. Subsequently, the synthesized Terp-2TH was subjected to thorough characterization using mass spectrometry to validate its structure. The obtained mass spectrum of Terp-2TH closely resembled the reported mass spectrum in the literature, providing compelling evidence for the accurate confirmation of its structure (Figure S1, ESI†). The mass spectrum exhibited a prominent peak corresponding to the molecular ion [M + H]^+^ at *m*/*z* 436.1254, consistent with the expected molecular weight of Terp-2TH (*m*/*z* 435.1154). The purified Terp-2Th was used to synthesize ZnTerp-2TH, as shown in Scheme 1. The ^1^H NMR spectrum of ZnTerp-2TH displayed two distinct singlet signals owing to the presence of two -NH protons at δ 9.08 and 9.11 ppm, respectively. The aromatic protons were observed within the range of δ 7.85–8.94 ppm. Additionally, a singlet peak resonated at δ 6.53 ppm, which corresponds to the ethylene proton conjugated between the terpyridine and 2TH ring (Figure S2, ESI†). The ^13^C NMR spectrum exhibited characteristic peaks at δ 165.51 ppm and 182.88 ppm for the -C=O and -C=S carbons of 2TH unit, respectively. The aromatic carbons resonated between δ 121.54 and 148.86 ppm. Further, the ethylene carbon was observed at δ114.89 ppm, whereas the other 2TH carbon was observed at 119.45 ppm (Figure S3, ESI†).

### 2.2. Thermal Analysis

**Figure 2:**
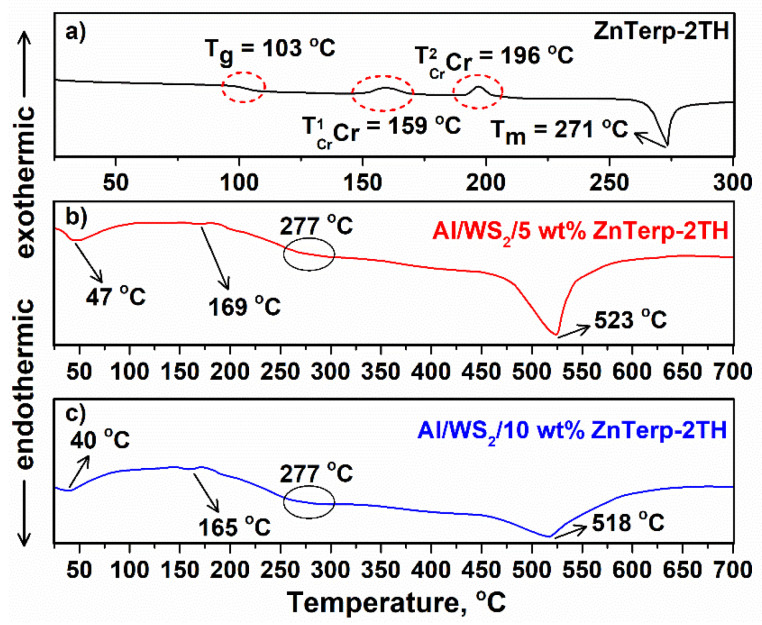
DSC curves of (**a**) ZnTerp-2TH, (**b**) Al/WS_2_/5 wt.% ZnTerp-2TH and (**c**) Al/WS_2_/10 wt.% ZnTerp-2TH composites.

The thermal phase transition behavior of ZnTerp-2TH and the microstructures of Al/WS_2_/5 and 10 wt.% ZnTerp-2TH composites were investigated by DSC from 25 °C to 300 °C and from 25 °C to 700 °C, respectively, applying a slow heating rate of 0.17 °C s^−1^ (10 °C min^−1^) under the breath of nitrogen. The DSC curves of pure ZnTerp-2TH and Al/WS_2_/5 and 10 wt.% ZnTerp-2TH composites are depicted in Figure 2. In Figure 2a, four thermal effects can be seen and the glass transition temperature (T_g_) was 103 °C and two crystallization temperatures (T_Cr_) were 159 and 196 °C, respectively, which were due to the precipitation and dissolution of organic rings and the result of the evaporation of bound hydroxyl [25]. The curve showed a strong and sharp endothermic peak at 271 °C, which corresponds to the melting point (T_m_) of ZnTerp-2TH. Exothermic and endothermic effects related to the formation and dissolution of Al/WS_2_/ZnTerp-2TH composites occurred, and the melting temperature was found to decrease with increasing ZnTerp-2TH content. The endothermic peaks at about 47 and 40 °C were attributed to the release of the physically adsorbed water and chloroform solvent within the structure from the samples [26]. Small peaks were observed at 169 and 165 °C, which may indicate the disintegrate chloroform and water vapor [27]. Furthermore, the endothermic peak appearing at about 277 °C is attributed to decomposition of the Zn derivative when the melting point of the ZnTerp-2TH component is close to 271 °C [28]. Moreover, the last main exothermic peaks at 523 °C and 518 °C are due to the stochastic chain deformation during the process, and in particular, are believed to be the result of the melting of Al (Figure 2b,c) [29] since the oxidation of WS_2_ may entail the formation of sulfides as the melting point [30,31]. In addition, it was found that at about 518 and 523 °C, the following reductive reaction between Al and WS_2_ can be initiated [10].

### 2.3. Study of X-ray Diffraction (XRD)

**Figure 3:**
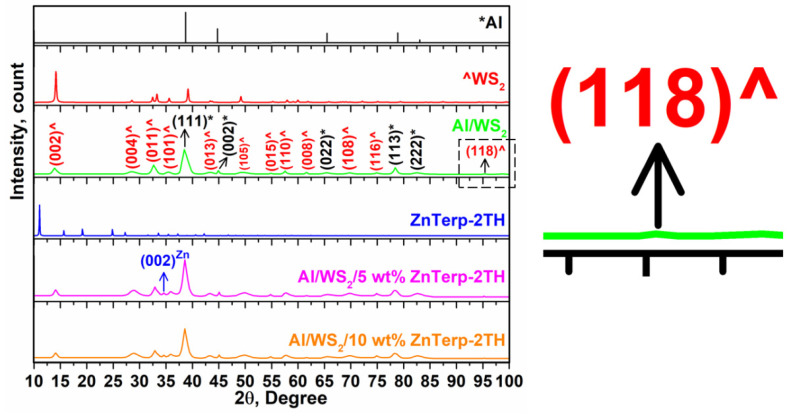
X-ray diffraction of Al/WS_2_, ZnTerp-2TH and Al/WS_2_/ZnTerp-2TH composites. (* Al and ^ WS_2_).

The schematic XRD pattern of Al from X’Pert and X-ray diffraction of WS_2_ and composites are illustrated in Figure 3. It can be seen that the diffracted planes (111), (002), (022), (113) and (222) at 2θ = 38.79°, 44.72°, 65.45°, 78.89° and 83.22° correspond to pure Al in a range of 2θ equal to 10–100° (JCPDS; 01-085-1327) [32,33]. In addition, the XRD pattern of WS_2_ shows the main diffraction plane (002) at 14.20° and (011) at 32.62°, which are characteristic of the hexagonal structure [34,35]. Furthermore, these peaks in the Al/WS_2_ XRD pattern confirmed the successful existence of WS_2_. Moreover, the XRD pattern of ZnTerp-2TH is shown and proved that this compound was crystalline and no amorphous phase or broad peaks were observed [36]. Further, the crystal size of the composites was calculated using the modified Scherrer equation [37] and values of 34, 26 and 27 nm were obtained for Al/WS_2_, Al/WS_2_/5 wt.% ZnTerp-2TH and Al/WS_2_/10 wt.% ZnTerp-2TH, respectively. In fact, the ZnTerp-2TH nanoparticles act as a barrier against grain boundary movement and prevent grain growth during the sintering process, which is why the nanocrystal size of the samples composed of ZnTerp-2TH was smaller than that of Al/WS_2_ and this issue is also discussed by other researchers in the literature [38,39]. It is worth noting that the XRD of the composites corresponded to the Al and WS_2_ patterns and the phases were similar. The difference between the diffraction patterns was that the intensity of the spectrum decreased with increasing ZnTerp-2TH content, as can be seen that the crystallite size of the composites composed of ZnTerp-2TH was smaller than that of Al/WS_2_ due to the existence of broader peaks in the Al/WS_2_ pattern. Additionally, the small effect of ZnTerp-2TH in the pattern at 2θ = 34.51° can be seen with corresponding Miller indices (002), which can be attributed to the content of ZnTerp-2TH in the synthesis of these composites. Additionally, this indicates that the temperature reached during the reaction was sufficient to induce ZnTerp-2TH in the composites. Moreover, the absence of reaction peaks confirms that the reaction for the preparation of Al/WS_2_/ZnTerp-2TH composites is completed within T = 250 °C at 2 h.

### 2.4. Fourier Transform Infrared (FTIR) Analysis

**Figure 4:**
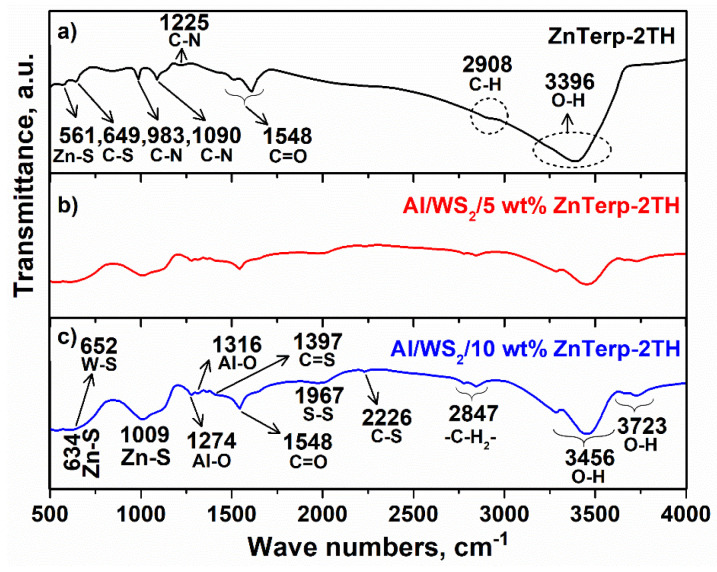
FTIR spectrum of (**a**) ZnTerp-2TH, (**b**) Al/WS_2_/5 wt.% ZnTerp-2TH and (**c**) Al/WS_2_/10 wt.% ZnTerp-2TH composites.

Figure 4 shows the FTIR spectrum of ZnTerp-2TH and Al/WS_2_/ZnTerp-2TH composites. It was noticeable that the FTIR spectra of the composites consisted of similar bonds since there were no impurities in the phase transitions as detected by XRD, and the difference was related to the intensity of transmission due to the content of ZnTerp-2TH. The broad peaks from 3008 to 3800 cm^−1^ were assigned to the O-H stretching vibration in all samples [40]. The weak bands at ~2908 and 2847 cm^−1^ were characteristic absorbance peaks of C-H and -C-H_2_-, respectively, as described in [41]. Furthermore, according to Figure 4a, the bands at 983, 1090, 1225 and 1548 cm^−1^ were assigned to the C-N bond, and the wavenumber at 1548 cm^−1^ was also associated with C=O [42]. The stretching band of the Zn-S group appeared at about 561 cm^−1^ in the ZnTerp-2TH spectra, which was confirmed according to reference [40]. Additionally, the band at 649 cm^−1^ was attributed to the C-S group in the ZnTerp-2TH structure [40]. In Figure 4b,c, for the composites, the absorption peaks at 634, 652 and 1009 cm^−1^ were attributed to the Zn-S lattice, which originated from the resonance interaction between the vibrational modes of the sulfide ions in the crystal [43]. In addition, at the wavenumbers of 1274 and 1316 cm^−1^, the bonds between Al and O groups were indicated, which could be attributed to the content of AlCl_3_ as the precursor and main component of the composite matrix [44]. Further, the band group appeared at about 1967 cm^−1^ for the S-S group [45].

### 2.5. Investigation of Morphology and Energy-Dispersive X-ray (EDX) Analysis

**Figure 5:**
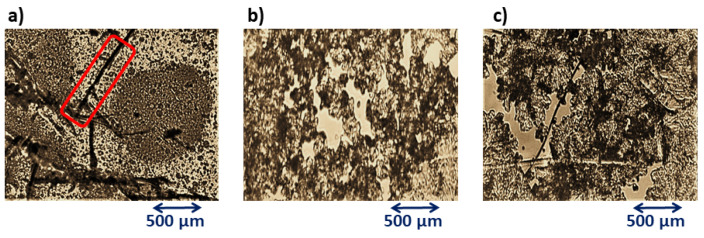
Optical microscope images of (**a**) Al/WS_2_, (**b**) Al/WS_2_/5 wt.% ZnTerp-2TH and (**c**) Al/WS_2_/10 wt.% ZnTerp-2TH composites.

The particles induce crystallite nucleation and the growth of 5 and 10 wt.% ZnTerp-2TH as reinforcement on the surface of Al/WS_2_, which prevents the formation of more vertical cracks and holes, as shown in Figure 5 (200× magnification) by the optical microscope. It is clear that several cracks and holes can be seen on the surface of Al/WS_2_, while these imperfections were covered and healed when ZnTerp-2TH was used, regardless of the ZnTerp-2TH content. In Figure 5c, a small crack can be seen, but the main location of the formed voids in front of the cracks and the micromechanism of the fracture did not exist. However, according to Figure 5a, the micromechanism of the fracture is ductile and dimples are clearly visible. Therefore, the mechanical properties of Al/WS_2_ composites can be improved compared to Al and WS_2_ alone, but the corrosion cannot be prevented. It is worth noting that Figure 5b shows the enlarged ZnTerp-2TH rich precipitates in the matrix, which destroyed the preferential sites for the formation of the voids. Thus, it could be argued that the enlargement of ZnTerp-2TH precipitates leads to non-premature failure because (1) the mechanical properties are improved and (2) preferential sites for the formation of voids are not available [46,47].

**Figure 6:**
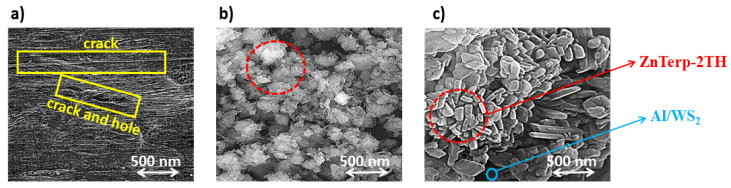
SEM images of (**a**) Al/WS_2_, (**b**) Al/WS_2_/5 wt.% ZnTerp-2TH and (**c**) Al/WS_2_/10 wt.% ZnTerp-2TH composites.

To investigate the distribution of nanoparticles in the composites, micrographs of scanning electron microscopy (SEM) were used, which are shown in Figure 6a–c for Al/WS_2_, Al/WS_2_/5 wt.% ZnTerp-2TH and Al/WS_2_/10 wt.% ZnTerp-2TH, respectively. Furthermore, the average diameter of the composite nanoparticles is estimated to be <40 nm.

Taking into account Figure 6, a crack with a length of more than 200 nm was observed in Al/WS_2_ when these cracks were reduced and disappeared by the addition of ZnTerp-2TH. According to the SEM sectional image of Al/WS_2_, there are intermetallic phase regions. Also, the continuous and consecutive cracks and holes were clearly observed at the grain boundary because WS_2_ diffused into the grains. The addition of ZnTerp-2TH as a reinforcement (Figure 6b,c) reduced the cracks and holes, and with increasing ZnTerp-2TH content, the nucleation and growth were enhanced. This process can help to improve the mechanical properties while reducing corrosion and increasing photocatalytic properties because the large agglomeration occurred to reduce the surface’s energy [48]. Moreover, the ZnTerp-2TH nanoparticles were densely arranged on the surface and developed a pore structure between the nanoparticles, which promoted the adsorption and degradation of the composites in the photocatalytic features [49,50]. This shows that the ZnTerp-2TH particles penetrated into Al/WS_2_ and supported it all around like a coating, which directly affects the corrosion and photocatalytic properties of the composites. Furthermore, an EDX analysis of these samples proves the presence of O, C, Cl, W, S, Al and Zn in the synthesized specimens and the amount values extracted from the EDX analysis are tabulated in Table 1. It should be mentioned that the absence of extra elements indicates that no significant impurities were present in the specimens and the values correspond to the content of the constituents during the preparation of the composites. According to this table, Cu was used as a reference, and with the increase in the weight percentage of ZnTerp-2TH, the values of Al and W decreased due to the particle distribution and the linkage of grain boundaries. In addition, the EDX analysis shows that Cl originates from AlCl_3_ as a precursor of Al/WS_2_ synthesis.

### 2.6. Corrosion Behavior

**Figure 7:**
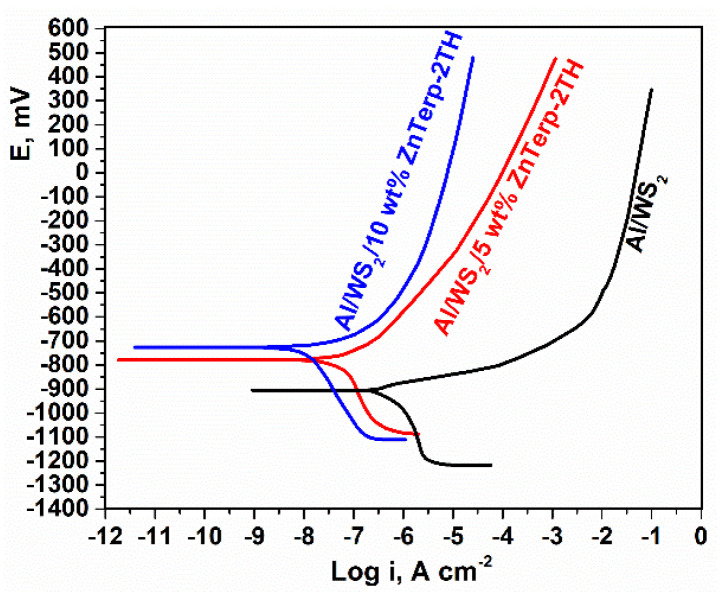
Potentiodynamic polarization curves of Al/WS_2_, and Al/WS_2_/ZnTerp-2TH composites.

The results of the corrosion test of the samples immersed in 3.5 wt.% aqueous NaCl solution for 10 h are shown in Figure 7 and Table 2, respectively. Moreover, no sudden increase in the current density of the samples was observed, indicating the passive nature of the prepared samples [51] when WS_2_ can also reduce the corrosion of Al. From the polarization curves, the current densities of the samples composed of ZnTerp-2TH are lower than those of Al/WS_2_ in the cathodic and anodic regions. These results indicate the reduced cathodic and anodic reaction rates of Al/WS_2_/ZnTerp-2TH composites compared to Al/WS_2_ alone. To investigate the corrosion behavior, the Stern–Geary Equation (1) was used as follows,

(1) $$I_{\mathrm{corr}}=\frac{1}{2.303R_{P}}\left(\frac{\beta_{a\times }\beta_{c}}{\beta_{a+}\beta_{c}}\right).$$

In this question, $I_{\mathrm{corr}}$ is the corrosion current density, $\beta_{a}$ is the slope of the anodic branch of the diagram, $\beta_{c}$ is the slope of the cathodic branch of the diagram and $R_{P}$ is the polarization resistance obtained by calculating the slope of the polarization diagram around an inflection point. The electrochemical polarization curves show that the corrosion potential (E_corr_) of Al/WS_2_/10 wt.% ZnTerp-2TH is −724 mV, which is 181 mV higher than that of the Al/WS_2_ sample (−905 mV), indicating the thermodynamic stability of this composition. It is also found that the E_corr_ of Al/WS_2_/5 wt.% ZnTerp-2TH is −773 mV, indicating that both composites have a more positive E_corr_ than Al/WS_2_. In addition, a significant decrease in I_corr_ to 5 uA cm^−2^ was observed for the composite with 10 wt.% ZnTerp-2TH, which is the better sample in preventing corrosion [52,53]. In addition, Al/WS_2_/10 wt.% ZnTerp-2TH showed a significant reduction not only of I_corr_ but also of E_corr_ toward the positive potential, confirming the improvement in the corrosion resistance of composites [54]. These results confirm that ZnTerp-2TH acts as a barrier and effectively resists the penetration of aggressive ${\mathrm{Cl}}^{-}$ ions into the composite. In particular, Al/WS_2_ wearing ZnTerp-2TH and also an increase in Zn derivative content lead to a decrease in I_corr_ values, which can be attributed to the formation of a relatively uniform distribution of ZnTerp-2TH particles as a cover that could act as a barrier against the penetration of corrosive ions, which is further confirmed by the surface and SEM morphology of this type of composite. This can be attributed to the sealing of holes and cracks in the specimens, which prevents the penetration of corrosive substances into the Al/WS_2_ substrate, and ZnTerp-2TH can have a positive effect on reducing the ratio of corrosion in Al/WS_2_.

The Zn complex extract as an inhibitor leads to a change in the slopes of the polarization curves, a decrease in the corrosion current density and an increase in the corrosion potential by interfering with the cathodic and anodic reactions. The presence of the inhibitor leads to a significant shift of the cathodic branches to a lower extent in the anodic branches of the polarization curves; therefore, ZnTerp-2TH could be classified as a mixed-type inhibitor with predominant cathodic activity. Moreover, ZnTerp-2TH as a ligand can form complex compounds due to its chelating effect. It is noteworthy that the inhibitory effect of ZnTerp-2TH was attributed to the formation of insoluble complex compounds in association with the metal cations. Aromatic compounds whose structure contains a delocalized π-electron system are susceptible to the delocalization of electrons in the phase, especially a ring with carbon. It is the delocalization of π-electrons that stabilizes the molecule.

## 3. Experimental

### 3.1. Synthesis of ZnTerp-2TH

A 20 mL methanol solution containing Terp-2TH (0.115 mmol) and ZnCl_2_ (0.563 mmol) was heated under reflux for 48 h and then allowed to cool to room temperature. The resulting yellow precipitate was separated by filtration, washed with cold ethanol and diethyl ether, and dried under ambient air conditions (Scheme 1). Yellow powder had a 67% yield. Melting point: 271 °C. ^1^H-NMR (DMSO-*d_6_*) (ppm): δ 9.11 (s, 1H), 9.08 (s, 1H), 8.93–8.94 (d, *J* = 8.0 Hz, 2H), 8.83–8.84 (d, *J* = 5.2 Hz, 2H), 8.30–8.33 (d, *J* = 7.8 Hz, 4H), 7.85–7.88 (t, *J* = 9.1 Hz, 6H), 6.53 (s, 1H); ^13^C-NMR (DMSO-*d_6_*) (ppm): δ 182.88 (-C=S), 165.51 (-C=O), 121.54–148.86 (aromatic carbon), 119.45 (2TH carbon), 114.89 (ethylene carbon); ESI-MS: calculated for C_50_H_34_N_10_O_2_S_2_Zn (*m*/*z*): 934.1599; found: 932.800 [M − 2]^+^.

### 3.2. Synthesis of Al/WS_2_ and Al/WS_2_/ZnTerp-2TH Composites

Due to the simple and inexpensive method for the synthesis of metallic composites, the composite Al/WS_2_ was prepared by a hydrothermal method, and the synthesis pathway of Al/WS_2_/ZnTerp-2H is shown in Figure 1. According to this method, (1) 5 mL of AlCl_3_ was dispersed in methanol solution (2M), and 30 wt.% WS_2_ was added under ultrasound for 30 min. (2) 6 mL of glycerol, sodium hexametaphosphate (SHMP) and deionized water (dropwise) were mixed at 80 °C for 20 min at a speed of 350 rpm with the aim of homogenization by ultrasonication. (3) The miscible component was autoclaved at 150 °C for 4 h and then gradually cooled to 25 °C. (4) The sample was separated by centrifugation at 8000 rpm for 15 min. (5) The product was washed 5 times with methanol. (6) The sample was dried at 80 °C for 4 h. (7) To remove volatile oxidizing substances, the samples were calcined at 300 °C for 1 h. (8) Due to the decomposition and transformation of this type of composite and taking into account the sensitivity to temperature treatments, spark plasma sintering (SPS) was considered and the sample was placed in a graphite mold for 5 min with pressure and temperature values of 50 MPa and 500 °C simultaneously.

The newly synthesized compound ZnTerp-2H was used to improve the physical and corrosive properties. (9) Considering 5 and 10 wt.%, ZnTerp-2H was dissolved in chloroform and added to the composite Al/WS_2_ at a temperature of 100 °C and stirrer speed of 350 rpm. (10) Finally taking into account the differential scanning calorimetry (DSC) of ZnTerp-2H, the appropriate temperature for the synthesis of Al/WS_2_/ZnTerp-2TH composites was selected 250 °C below the melting point of ZnTerp-2TH for 2 h.

## 4. Conclusions

Taking into account Al/WS_2_ as matrix and ZnTerp-2TH as reinforcement, a new type of corrosion-resistant Al/WS_2_/ZnTerp-2TH composites was prepared in this study. A thermal analysis revealed melting points of 271, 523 and 518 °C for ZnTerp-2TH, Al/WS_2_/5 wt.% ZnTerp-2TH and Al/WS_2_/10 wt.% ZnTerp-2TH composites, respectively. An X-ray diffraction analysis proved that there were no impurities between the components, and the modified Scherrer method was used to determine crystallite sizes of 34, 26 and 27 nm for Al/WS_2_, Al/WS_2_/5 wt.% ZnTerp-2TH and Al/WS_2_/10 wt.% ZnTerp-2TH, tandemly. The FTIR spectrum showed the bond Zn-S from the reinforcement and Al-O, C=O, C-S and S-S groups. Moreover, the morphology was discussed in detail when the ZnTerp-2TH nucleated and grew on the surface of Al/WS_2_ that this phenomenon led to the prevention of the corrosion of Al/WS_2_. An EDX analysis proved that all compositions were derived from the precursors in the synthesis process. The composite Al/WS_2_/10 wt.% ZnTerp-2TH showed better corrosion results than the other counterpart when E_corr_ and I_corr_ were reached in tandem to −724 mV/decade and 5 uA cm^−2^. Overall, the design and synthesis of ZnTerp-2TH as reinforcement improves the corrosion resistance ratio.

## Figures and Tables

**Table 1:** Extracted values from EDX analysis.

Al/WS_2_		Al/WS_2_/5 wt.% ZnTerp-2TH		Al/WS_2_/10 wt.% ZnTerp-2TH	
Element	Weight, %	Element	Weight %	Element	Weight %
**O**	27.99	**O**	31.08	**O**	29.15
**C**	32.29	**C**	34.98	**C**	34.16
**Cl**	0.86	**Cl**	0.37	**Cl**	0.24
**W**	9.76	**W**	5.11	**W**	7.09
**S**	3.73	**S**	5.09	**S**	6.17
**Al**	24.04	**Al**	18.02	**Al**	16.14
**Cu**	1.33	**Zn**	3.77	**Zn**	5.81
		**Cu**	1.58	**Cu**	1.24

**Table 2:** Extracted values from corrosion analysis.

Samples	E_corr_, (mV/Decade) vs. SCE	β_a_, (mV/Decade) vs. SCE	β_c_, (mV/Decade) vs. SCE	I_corr_, µAcm^−2^
**Al/WS_2_**	−905	765	−585	29
**Al/WS_2_/5 wt.% ZnTerp-2TH**	−773	352	−466	14
**Al/WS_2_/10 wt.% ZnTerp-2TH**	−724	661	−326	5

## Data Availability

Data sharing is not applicable.

## Supplementary Materials

The following supporting information can be downloaded at: https://www.mdpi.com/article/10.3390/molecules28217290/s1.
