# Supplementary material for: Significance of Zn Complex Concentration on Microstructure Evolution and Corrosion Behavior of Al/WS2
Source: Molecules. 2023 Oct 27;28(21):7290. doi: 10.3390/molecules28217290 (PMC10650769; doi:10.3390/molecules28217290)
Supplement: Supplementary file 1 [file molecules-28-07290-s001.zip › molecules-2660141-supplementary.pdf]

molecules

MDPI

Article

Significance of Zn Complex Concentration on Microstructure Evolution and Corrosion Behavior of Al/WS_2

Pratiksha P. Gawas [1], Praveenkumar Pandurangan [1], Marzieh Rabiei [2], Arvydas Palevicius [2], Andrius Vilkauskas [2], Giedrius Janusas [2], Mozhgan Hosseinnezhad [3], Reza Ebrahimi-Kahrizsangi [4], Sohrab Nasiri [5,6,*], Jean Michel Nunzi [6] and Venkatramaiah Nutalapati [1,*]

1 Functional Materials Laboratory, Department of Chemistry, Faculty of Engineering and Technology, SRM Institute of Science and Technology, SRM Nagar, Kattankulathur 603203, India; pratiksha.gws@rediffmail.com (P.P.G.); pp9547@srmist.edu.in (P.P.)

2 Faculty of Mechanical Engineering and Design, Kaunas University of Technology, Studentu Street 56, LT 51373 Kaunas, Lithuania; rabieimarzieh7@gmail.com (M.R.); arvydas.palevicius@ktu.lt (A.P.); andrius.vilkauskas@ktu.lt (A.V.); giedrius.janusas@ktu.lt (G.J.)

3 Department of Organic Colorants, Institute for Color Science and Technology, Tehran 1668836471, Iran; hosseinnezhad-mo@icrc.ac.ir

4 Advanced Materials Research Center, Department of Materials Engineering, Najafabad Branch, Islamic Azad University, Najafabad Isfahan 85141-43131, Iran; rezaebrahimi@iaun.ac.ir

5 Faculty of Mechanical Engineering, Optical Measurement Laboratory, Kaunas University of Technology, Studentu Street 56, L-116, LT 51373 Kaunas, Lithuania

6 Department of Physics, Engineering Physics & Astronomy, Queens University, Kingston, ON K7L-3N6, Canada; nunzijm@queensu.ca

* Correspondence: sohrab.nasiri2017@gmail.com or sohrab.nasiri@ktu.edu (S.N.); nvenkat83@gmail.com (V.N.); Tel.: +370-65586329 (S.N.); +91-8328217144 (V.N.)

Materials and experiments

The chemical components and solvents were aluminum chloride ($AlCl_3$), methanol, tungsten sulfide (WS_2), sodium hexametaphosphate (SHMP), glycerol ($C_3H_8O_3$), Sigma Aldrich. The Bruker 500 MHz spectrometer used to record the NMR (^{1}H and ^{13}C), with the chemical shifts (δ) and coupling constants (J) reported in parts per million (ppm) and Hz respectively. Agilent 6230B Time of Flight (TOF) technology used to acquire Mass spectra. In the synthesis route, the stainless steel autoclave 10L, microcentrifuge CF 48, furnace E5CK-AA1-302 (Snol 6, 7/1300), spark plasma sintering (SPS) A-8000 stainless steel double-walled vacuum chamber with the ability to cool were used. For thermal analysis, differential scanning calorimetry (DSC) STA-BAHR, and for X-ray diffraction (XRD), a Philips XRD diffractometer with Cukα radiation at 40 KV, 30 mA, a step size of 0.05° (2θ) and a scan rate of 1o/min were considered. X'Pert software (version 4.9.0) was used for qualitative analysis and to indicate the width of diffraction peaks (rad, β) at full width half maximum (FWHM) at different 2θ values according to the location of the peaks. In the modified Scherrer method for calculating the crystallite size of the samples, Lnβ was plotted versus Ln(1/cosθ) and the least squares method was applied; the intercept gives Ln(Kλ/L), from which a single value for L can be obtained. $\frac{K\,\lambda Cu_{k\alpha 1}}{L} = e^{(\text{intercept})}$, in the equation K is the shape factor (K=0.89), $\lambda_{Cuk\alpha 1}$ = 0.15405 nm, L is the crystal size, and the intercept is related to the linear equation. FTIR (Perkin Elmer) was used to study the bonding of groups, and Nikon-H eclipse optical microscope and scanning electron microscopy (SEM) VEGA\\TESCAN-LMU were also used to study the morphology of specimens. To evaluate the corrosion, the potential polarization method A 2273 potentiostat/galvanostat was used to investigate the corrosion behavior of the samples. To perform this test, after setting the parameters, the instrument automatically reads the open circuit potential (OCP) and starts cycling the potential. Further, all experiments were performed at room temperature. A calomel electrode (saturated potassium chloride) (SCE) served as the

reference electrode. Electrochemical testing was performed at room temperature in NaCl solution using an electrochemical apparatus (model PARstat 2273). A three-electrode cell was used with a graphite of suitable area as the counter electrode and a saturated calomel electrode (SCE, Radiometer Copenhague) as reference electrode. Potentio dynamic tests were performed in the range of -0.25 to 0.7 V versus open circuit potential with a scan rate of 0.001 Vs^{-1}.

Structural Characterization

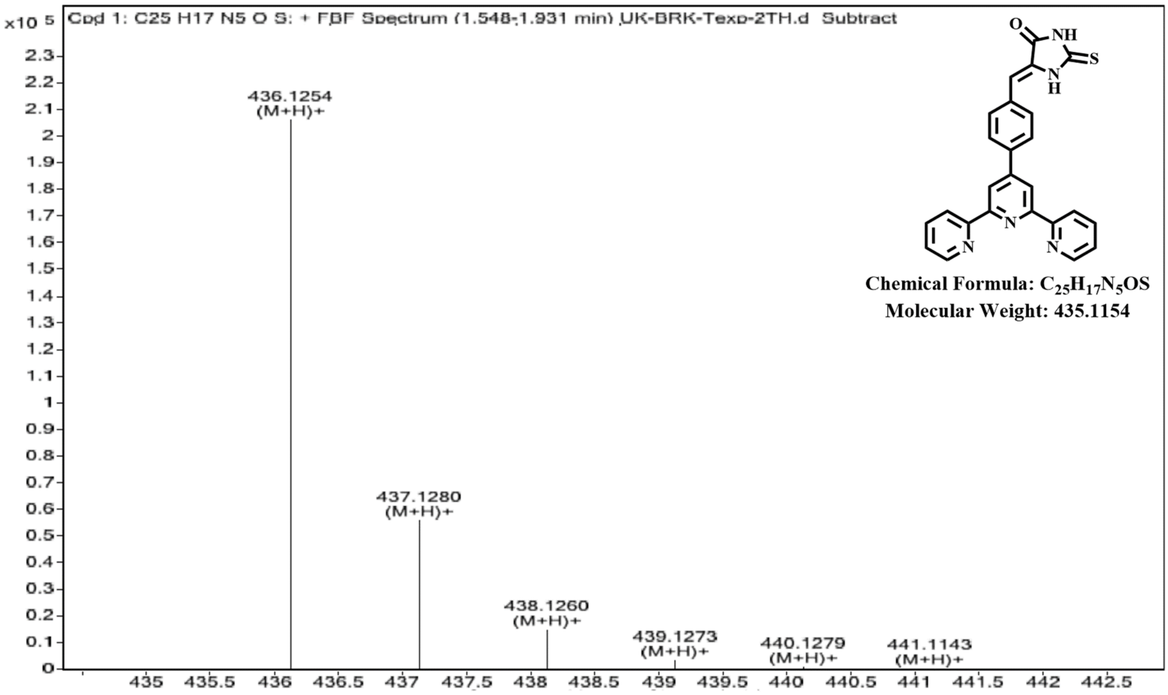

Figure S1. HR-MS spectra of Terp-2TH.

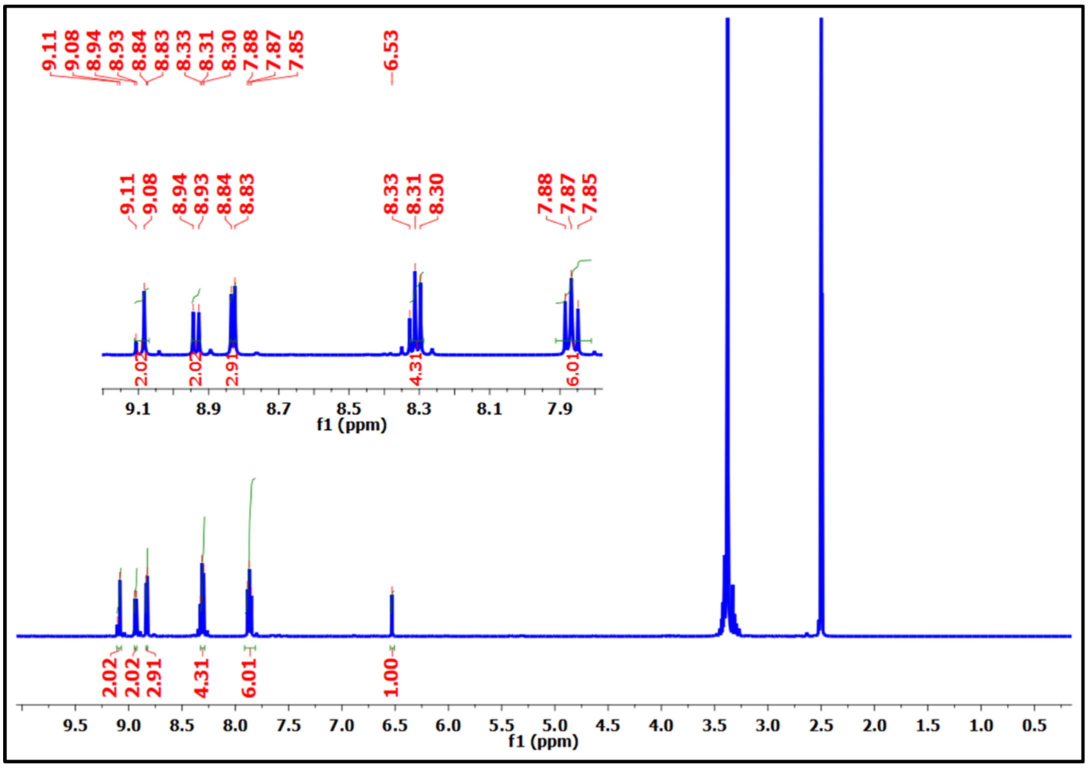

Figure S2. ^{1}HNMR spectra of ZnTerp-2TH in DMSO-d_6.

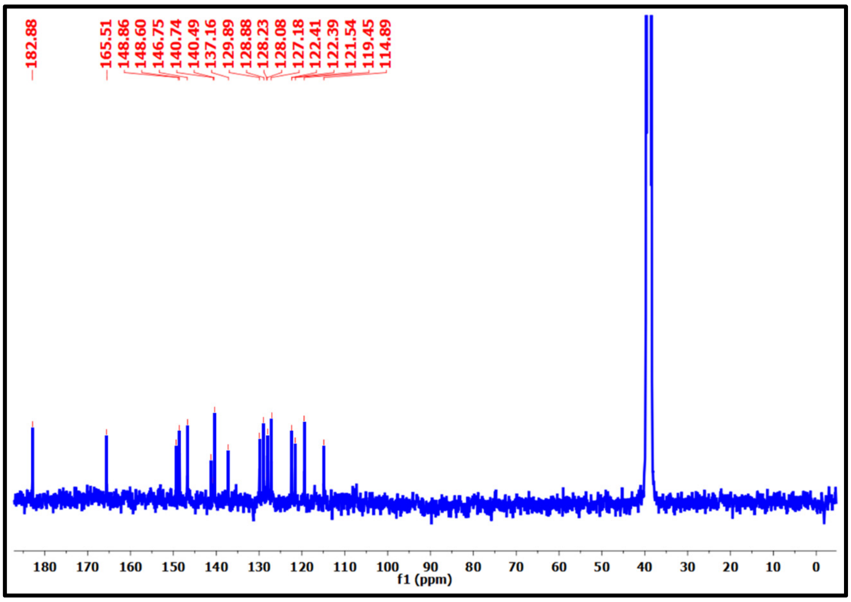

Figure S3. ^{13}CNMR spectra of ZnTerp-2TH in DMSO-d6.

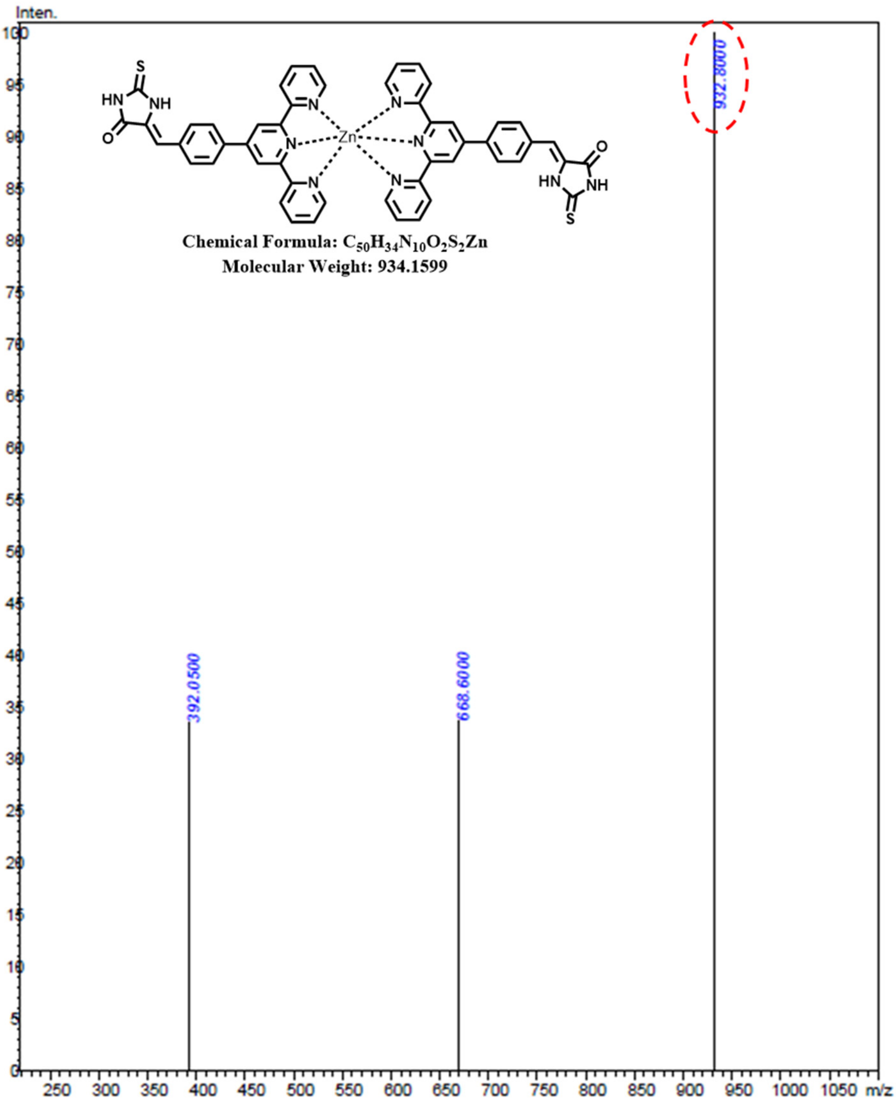

Figure S4. HR-MS spectra of ZnTerp-2TH.
